# Chronomedicine Insights: Evaluating the Impact of Time-Restricted Meal Intake on Lipid Profile Parameters Among Individuals With Type 2 Diabetes in Northern India

**DOI:** 10.7759/cureus.56902

**Published:** 2024-03-25

**Authors:** Smriti Rastogi, Narsingh Verma, Gourav Raghuwanshi, Dileep Kumar Verma, Virendra Atam

**Affiliations:** 1 Physiology, King George's Medical University, Lucknow, IND; 2 Physiology, People's College of Medical Sciences and Research Centre, Bhopal, IND; 3 Internal Medicine, King George's Medical University, Lucknow, IND

**Keywords:** chronobiology, endocrinology, metabolism, diabetes, lipid profile

## Abstract

Introduction: Time-restricted meal intake (TRM) has shown potential benefits such as enhanced insulin sensitivity, lowered blood sugar levels, and possible weight loss in individuals with type 2 diabetes mellitus (T2DM). Our study aimed to investigate the impact of TRM on lipid profile parameters such as total cholesterol (TC), triglycerides (TG), high-density lipoprotein (HDL), low-density lipoprotein (LDL), and very low-density lipoprotein (VLDL) in fasting conditions in T2DM patients.

Methods: In total, 400 patients from the endocrinology department at King George's Medical University (KGMU), Lucknow were enrolled in this study, adhering to the guidelines of the American Diabetes Association (ADA). Male and female patients with recently diagnosed T2DM (in the past five years), aged between 25 to 60 years, on oral anti-diabetic therapy excluding insulin, expressing willingness to provide written consent and to adhere to TRM were included in the TRM group. It was a longitudinal study as diabetic dyslipidemia is primarily caused by insulin resistance and nutritional reasons and we wanted to assess the effect of TRM on lipid profile in T2DM patients. Patients were enrolled via simple random sampling using the random number table method (computerized). The TRM group had an early dinner at 7 pm whereas the control group was of non-TRM/late-night eaters. TRM group was given comprehensive guidance including strategies to manage hunger, permissible beverage options (water and prescribed medication) during the fasting period, and daily maintenance of a diary documenting their timing, type, and quantity of food intake which they were requested to bring fortnightly. Emphasis was placed on recording even minor dietary items consumed throughout the day. The TRM group consumed food ad libitum during a 12-hour eating window from breakfast at 7 am to dinner at 7 pm. Data distribution was non-parametric. Mann-Whitney U test compared TRM and control group using mean values at baseline and follow-ups. Analysis used GraphPad Prism 9.2.0 software (GraphPad Inc., La Jolla, CA). A p-value less than 0.05 (p < 0.05) was considered statistically significant.

Results: A total of 127 patients were lost to follow-up, resulting in 273 patients who completed the study. The mean value of TC in the TRM and non-TRM groups using the Mann-Whitney U test registered a highly significant p-value <0.0001 at 18 months, with a decrease of 14.17% from baseline in the TRM group and a decrease of 1.53% from baseline in the non-TRM group. The TRM group had a decrease of 24.75% in TG from a baseline value of 145.4±41.9, whereas the non-TRM group showed a decrease of 2.2% from a baseline value of 154.7±37.30 (p-value <0.0001). The TRM group showed an increase of 9.25% in HDL from a baseline value of 50.14±8.58; the non-TRM group showed an increase of 0.82% from a baseline value of 48.62±9.31 (p-value <0.0001). TRM group showed a decrease of 8.62% in LDL from a baseline value of 68.20±16.2 while the non-TRM group showed an increase of 1.54% from a baseline value of 65.38±19.3 (p-value <0.0002). The TRM group showed a decrease of 13.97% in VLDL from a baseline value of 32.20±18.7; the non-TRM group showed an increase of 4% from a baseline value of 30.16±24.2 (p-value <0.0001).

Conclusion: Our study's promising results underscore the potential of TRM as an effective strategy for managing dyslipidemia in individuals with T2DM, even over prolonged periods.

## Introduction

In India, an estimated 74 million adults have diabetes, with over 90% likely to experience atherogenic dyslipidemia; an additional 70 million with pre-diabetes may also be affected [1]. This alarming figure underscores the urgent need for understanding and addressing the factors contributing to the diabetes epidemic in the country. Among the multifaceted causes, lifestyle changes, genetic predisposition, and environmental factors play pivotal roles in the onset and progression of diabetes, particularly type 2 diabetes mellitus (T2DM). The delayed diagnosis and undertreatment of dyslipidemia in T2DM significantly contributes to heightened rates of cardiovascular disease (CVD) [2]. This highlights the urgency for early diagnosis and targeted treatment. Indian patients with T2DM often exhibit unique characteristics compared to Western populations, including earlier onset, a higher prevalence of metabolic syndrome features, and a more aggressive course of atherosclerotic cardiovascular disease (ASCVD) complications [3]. The distinct phenotype of the Indian population, characterized by elevated body fat mass despite normal weight or body mass index (BMI), underscores their predisposition to insulin resistance, dyslipidemia, and increased risk of T2DM [4]. Despite having a mean BMI within the obese range, nearly half of non-diabetic Indians exhibit dyslipidemia, with notably higher rates of atherogenic small, dense low-density lipoprotein (LDL) particles compared to their counterparts in the United States. Furthermore, the risk of coronary artery disease (CAD) among Indians is considerably higher, necessitating aggressive interventions to mitigate ASCVD risk factors [3,4].

In T2DM, dyslipidemia typically manifests as hypertriglyceridemia, low high-density lipoprotein cholesterol (HDL-C), and small dense LDL particles, with elevated non-HDL-C being a more accurate predictor of ASCVD risk [4]. Glycation-induced abnormalities in lipoprotein function further exacerbate dyslipidemia in diabetes. Despite achieving target LDL-C (low-density lipoprotein-cholesterol) levels, diabetes independently elevates cardiovascular event risk, necessitating comprehensive management of dyslipidemia alongside glycemic control [3].

The primary approach to managing insulin resistance and diabetes-related atherogenic dyslipidemia involves lifestyle modifications alongside statin therapy, with additional therapies considered necessary in conjunction with glycemic control. Dietary patterns play a crucial role in the development, progression, and management of T2DM. Time-restricted meal intake (TRM) has emerged as a promising dietary approach that has garnered significant attention in recent years. TRM involves restricting daily food consumption to a specific time window, typically ranging from 8-12 hours, and fasting for the remaining hours of the day [5]. This approach is grounded in the concept of aligning eating patterns with the body's natural circadian rhythms, which govern various physiological processes, including metabolism and energy regulation.

There is a lack of research specifically addressing the effects of TRM on lipid profiles in the Indian population, and studies with extended follow-up periods beyond one year are limited. By addressing this research gap, our study aims to provide valuable insights into the long-term benefits of TRM for T2DM patients in the Indian context. Understanding how TRM influences lipid profiles can contribute significantly to improving the management and treatment of T2DM. Therefore, the objective of our study was to assess the effect of TRM on lipid profile in fasting conditions in T2DM patients. We enrolled patients at baseline and assessed the lipid profile parameters at 6 months (1st follow-up), 12 months (second follow-up), and 18 months (third follow-up).

TRM and its role with respect to lipid metabolism

TRM refers to the practice of restricting the time window within which meals are consumed over a 24-hour period. To date, most TRM studies have utilized eating windows ranging from 4-12 hours without imposing caloric restrictions. Some researchers view TRM as a chrono-nutritional strategy [6,7]. The flexible nature of TRM protocols allows individuals to maintain their preferred eating patterns, potentially enhancing adherence and compliance with the dietary regimen. Additionally, aligning feeding and fasting cycles with the body's circadian rhythm-eating during the active phase and fasting during the resting phase positively impacts nutrient metabolism, hormonal regulation, and physiological processes, ultimately improving cardiometabolic health. Incorporating recent insights, TRM has emerged as a potential modulator of lipid metabolism and insulin sensitivity. Research suggests that aligning eating patterns with the body's circadian rhythm may positively impact glucose control and lipid levels, providing a promising avenue for diabetes management [8].

A number of studies have attributed the cardiometabolic benefits of TRM to the circadian rhythm and biological clocks, which influence glucose regulation, beta cell responsiveness, body composition, weight management, reduction of oxidative stress, and metabolic switching [9-13]. When fasting, the body is deprived of glucose, and after glycogen stores are depleted (typically within 12 hours of fasting), energy production shifts to alternative sources such as fatty acids and ketone bodies. This metabolic switch, often referred to as "flipping," is a crucial aspect of fasting-induced metabolic changes [14]. This metabolic shift involves increased fatty acid oxidation in various tissues, including the liver and skeletal muscle, to produce energy. Additionally, fat stores are broken down into fatty acids, which can be further metabolized to produce ketone bodies through a process called ketogenesis [15].

Ketone bodies, such as beta-hydroxybutyrate (BHB), acetoacetate, and acetone, serve as alternative fuel sources for tissues like the brain, heart, and skeletal muscle during periods of fasting or prolonged carbohydrate restriction. The utilization of ketones as an energy substrate reduces the reliance on glucose and helps preserve glycogen stores, especially in the brain, which is particularly sensitive to fluctuations in glucose availability.

Furthermore, the metabolic switch induced by fasting promotes autophagy, a cellular process that involves the degradation and recycling of damaged or dysfunctional cellular components. Autophagy plays a crucial role in cellular homeostasis, energy metabolism, and adaptation to stress conditions. The elongation of the fasting period during TRM leads to metabolic adaptations characterized by increased fatty acid oxidation, ketogenesis, and autophagy. These metabolic changes contribute to the utilization of stored fat for energy, maintenance of blood glucose levels, and cellular rejuvenation, which may underlie the observed metabolic benefits of TRM [16].

## Materials and methods

The study was conducted in the outpatient department (OPD) of the endocrinology department at King George's Medical University (KGMU), Lucknow. Ethical clearance was obtained from KGMU's institutional ethics committee (approval number 881/Ethics/R.Cell-18). The ethics committee met on 8th June 2018 and approved the study. The study design was longitudinal with a total duration of 18 months. The sample size was calculated using the prevalence of diabetes in India and totaled 400 patients. Simple random sampling using the random number table method (computerized) was used to enroll patients in each group. We devised this study to assess whether or not TRM had any effect on the lipid profile measured in fasting conditions in T2DM patients.

A strict selection criteria was followed for enrolling patients for the study. This study targeted patients within the age range of 25-60 years who were diagnosed with T2DM and were receiving care at the endocrinology department of KGMU, Lucknow. Eligible participants were required to be recently diagnosed T2DM patients (i.e. diagnosed with T2DM less than five years previously) and on a stable regimen of oral anti-diabetic medications, which included metformin, sulfonylurea, gliptin, or glitazones, but not insulin. The patients included were those who had no change in anti-diabetic treatment for at least one month prior to enrolment. Critical to their inclusion was their willingness to provide written informed consent and comply with the TRM plan which was integral to the study. Patients in the control group were also included based on the above inclusion criteria, the only difference being that they did not follow TRM at all. Individuals excluded from the study were those with any form of diabetes other than T2DM. Also, patients with significant renal impairment, indicated by a serum creatinine level exceeding 1.5mg/ml, or severe liver disease, where aspartate transaminase (AST) or alanine transaminase (ALT) levels were equal to or more than three times the normal limit were excluded from the study. Pregnant or lactating women, along with those planning to become pregnant, were also not considered for the study. Further, patients who had participated in another clinical trial in the previous 30 days or had a history of cardiovascular, hepatic, renal, psychiatric illnesses, or hemoglobinopathies were excluded. The use of sodium-glucose transport protein 2 (SGLT2) Inhibitors or glucagon-like peptide-1 receptor agonists (GLP 1 RA) were also a disqualifying factor, as was regular engagement in overnight shift work or excessive alcohol consumption. The patients diagnosed as having T2DM as per American Diabetes Association (ADA) guidelines by the physician in the OPD and those who met our selection criteria, were recruited to the study after obtaining written informed consent from each patient. 

The total duration of the study was from 19 December 2017 to 30 May 2022. The total number of patients recruited in the study from 19 June 2018 to 30 July 2019 was 400, however, 127 patients were lost to follow-up due to various reasons such as COVID-19, non-compliance, relocation, refusal to pick up calls and job transfers so the total number of patients who completed the study was 273. Patients were allocated into two groups, one group was the TRM group and the other was the late-night eater/non-TRM/control group. Among the enrolled patients, 134 patients were assigned to the TRM (early dinner at 7 pm) group, while 139 patients were allocated to the control/non-TRM/late-night eater group. We assessed age, height, weight, and the five parameters of fasting lipid profile namely total cholesterol (TC), triglycerides (TG), high-density lipoprotein (HDL), low-density lipoprotein (LDL), and very low-density lipoprotein (VLDL) in patients of T2DM. All the parameters were measured from baseline to a period of 18 months with three follow-ups, the first follow-up at six months, the second follow-up at 12 months, and the third follow-up at 18 months.

There were 134 patients in the TRM group with 54 (40.3%) females and 80 (59.7%) males and 139 patients in the control group with 60 (43.16%) females and 79 (56.84%) males. Patients were divided into the TRM and control groups based on their consent to adhere to an early dinner schedule. The TRM group agreed to have dinner at 7 pm, while the control group did not alter their meal timings and continued to have late-night dinner. The intervention involved counseling each patient in the TRM group about the benefits and protocols of early dinner, including dietary guidelines and coping strategies. The patient’s demographic information, detailed medical history, age, weight, and height were recorded immediately at their first visit to the OPD and this was designated as the baseline data. Body weight was measured using a digital scale to the nearest 0.1 kg. Patients were weighed without shoes and in light clothing. Height was measured to the nearest 1 cm using a stadiometer. Blood samples were collected after 12 hours of fasting for lipid profile assessment for each patient. A single intervention was done in the TRM group the timing of dinner was at or around 7 pm. Baseline parameters were recorded during the first visit to the OPD, and follow-up was done. In total, 134 patients aged 25-60 years were evaluated, educated, examined, and trained in circadian eating patterns. A proforma including the details of the patients was maintained along with a diet chart which was updated on a weekly basis (from Sunday to Monday) for all patients. All patients irrespective of their group in the study were on the standard conventional treatment of T2DM.

Patients received detailed dietary guidance and were instructed to maintain a food diary to document daily food intake. The diary included all consumed items, even small snacks (a piece of chocolate, candy, chewing gum tobacco, etc.) or beverages including the time, quantity, and type of food intake. Patients in the TRM group were allowed to eat ad libitum within a 12-hour eating window, starting from breakfast at 7 am to dinner at 7 pm, with only water and medications permitted outside this window. All patients were given strict instructions to bring the food diary fortnightly to the OPD so that compliance with the TRM plan could be ensured. Follow-up assessments were conducted at six-month intervals over 18 months to track changes in parameters and outcomes. Overall, the study implemented structured interventions and monitoring procedures to evaluate the effects of early dinner on body weight and lipid profile over an extended period. The TRM intervention focused on meal timing adjustment, supported by comprehensive counseling and dietary monitoring. The follow-up assessments also evaluated long-term outcomes and adherence to the intervention protocol.

In TRM studies conducted in human subjects, it remains a challenge to ensure the compliance and strict adherence of each patient to the TRM protocol. We have taken stringent methods to ensure the same in our study. Some measures like maintaining a detailed diet diary, and documenting timing, quantity, and food type intake, proved to be a crucial yet daunting aspect of our study. We ensured compliance with the 12-hour fasting protocol among participants in the TRM group through the recall method and regular monitoring. Throughout the 18-month study duration, patients were required to present their diet diaries fortnightly during OPD visits for counseling sessions. During these sessions, dietary records were reviewed meticulously, and patients were reminded to document even minor snacks, ranging from a piece of chewing gum or candy, to tobacco. Patients had to write the timing and quantity of breakfast, lunch, and dinner along with any other food item that they consumed during the course of the day. Failure to maintain or provide the diary led to removal from the study group. To monitor adherence further, patients received biweekly calls to assess any adverse effects from fasting, to address any problems of compliance with the 12-hour fasting regimen, and any other challenges faced in following the TRM schedule. Detailed guidance on managing hunger was provided to facilitate adherence to the TRM pattern. During the entire duration of our study, there was not a single incident of adverse effects due to TRM, and by using the recall method, strict monitoring, and regular phone calls we tried our best to ensure compliance but this was subject to the patient's subjectivity as well.

 Statistics

The normal distribution of the data was assessed, and it was found that our data did not follow a parametric distribution. Therefore, the Mann-Whitney U test (non-parametric test) was employed to compare the TRM and control groups at baseline and at each stage of follow-up using mean values. We deemed the results to be clinically significant if previously non-significant mean values at baseline became significant at the follow-up assessments. Data analysis was conducted using GraphPad Prism 9.2.0 software (GraphPad Software, San Diego, CA). A p-value less than 0.05 (p < 0.05) was considered statistically significant, and a two-tailed p < 0.05 was considered significant.

## Results

The mean age of the patients in the TRM group was found to be 47.17±10.22 years, and the mean age in the non-TRM group was 46.97±10.36 years. In the TRM group, the mean height (in meters) was 1.58±0.08, and in the non-TRM group, the mean height (in meters) was 1.59±0.10. Patients in the TRM group experienced a significant weight loss of 3.88 kg (5.45%) from their baseline weight of 71.1±12.38 kg. In contrast, the non-TRM group showed a smaller weight loss of 1.36 kg (1.77%). The mean of the weights was significantly different at the baseline between the two groups, but the difference became highly significant in subsequent follow-ups, signifying a greater reduction in weight in the case/TRM group.

Parameters measured

Total Cholesterol (TC)

**Figure 1:**
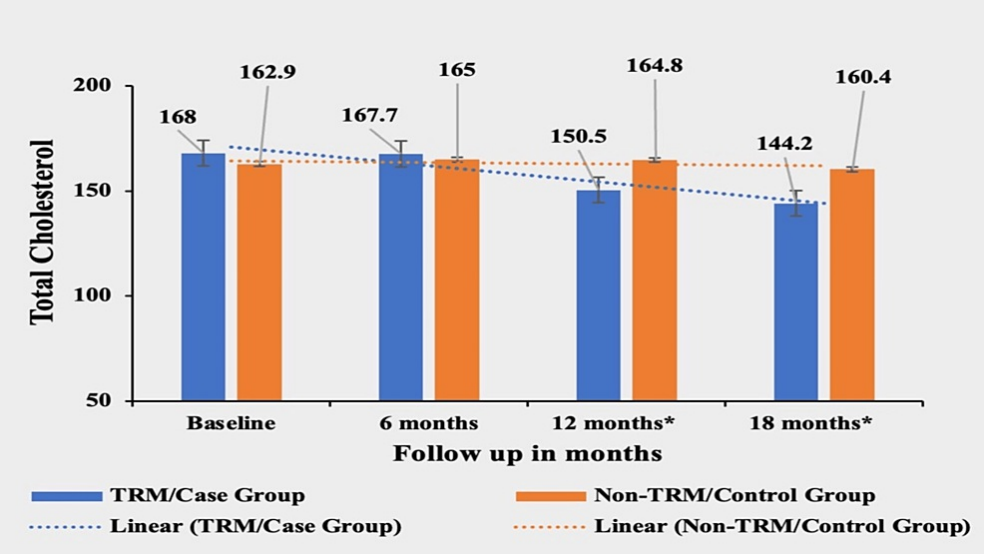
Comparative analysis between TRM/case group and non-TRM/control group on the basis of Total Cholesterol Columns represent the mean values of total cholesterol for TRM and non-TRM groups; error bars represent standard deviation (mean±SD). *p-value < 0.0001 TRM: time-restricted meal intake, non-TRM: non-time-restricted meal intake

Patients in the TRM group experienced a significant decrease of 14.17% in total cholesterol from their baseline value of 168±32.31. The mean value of TC in the first, second, and third follow-ups were 167.7±27.7, 150.5±29.3, and 144.2±27.4, respectively. In contrast, the non-TRM group showed a smaller decrease of 1.53% from their baseline value of 162.9±37.71, 165±34.97 in the first follow-up, 164.8±32.22 in the second follow-up and 160.4±31.03 in the third follow-up. The mean of the total cholesterol was not significantly different between the two groups, as the baseline p-value was 0.1157 and at the first follow-up, the p-value was 0.4738, but the difference became highly significant in subsequent follow-ups, with a p-value of <0.0002 at second follow-up and p-value of 0.0001 at third follow-up, signifying a greater reduction in cholesterol in the case/TRM group (Figure 1).

Triglycerides (TGs)

**Figure 2:**
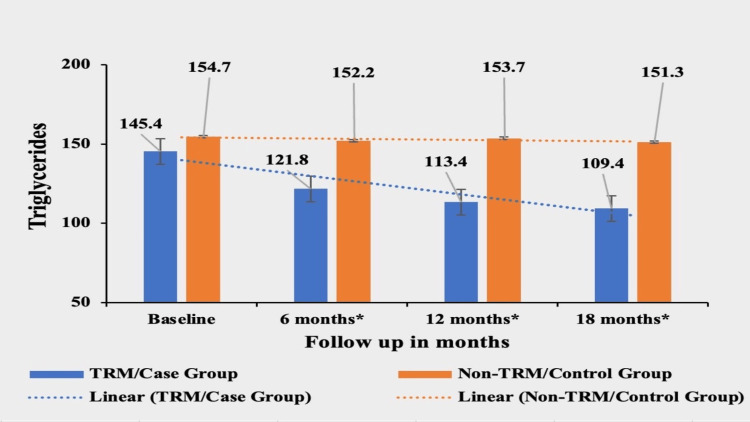
Comparative analysis between TRM/case group and non-TRM/control group on the basis of TGs Columns represent the mean values of TGs for TRM and non-TRM groups; error bars represent standard deviation (mean±SD). * p-value < 0.0001 TRM: time-restricted meal intake, non-TRM: non-time-restricted meal intake; TG: triglycerides

Patients in the TRM group experienced a significant decrease of 24.75% in TG from their baseline value of 145.4±41.9. The mean values of TGs in the first follow-up, second follow-up, and third follow-up were 121.8±25.7, 113.4±20.6, and 109.4±16.54, respectively. In contrast, the non-TRM group showed a smaller decrease of 2.2% from their baseline value of 154.7±37.30; 152.2±34.92 in the first follow-up, 153.7±33.89 in the second follow and 151.3±37.31 in the third follow-up. The mean of TGs was not significantly different at the baseline p value 0.0510 between the two groups, but the difference became highly significant in subsequent follow-ups, with p-values of <0.0001 at all three follow-ups, signifying a greater reduction in TG in the case/TRM group (Figure 2).

High-Density Lipoprotein (HDL)

**Figure 3:**
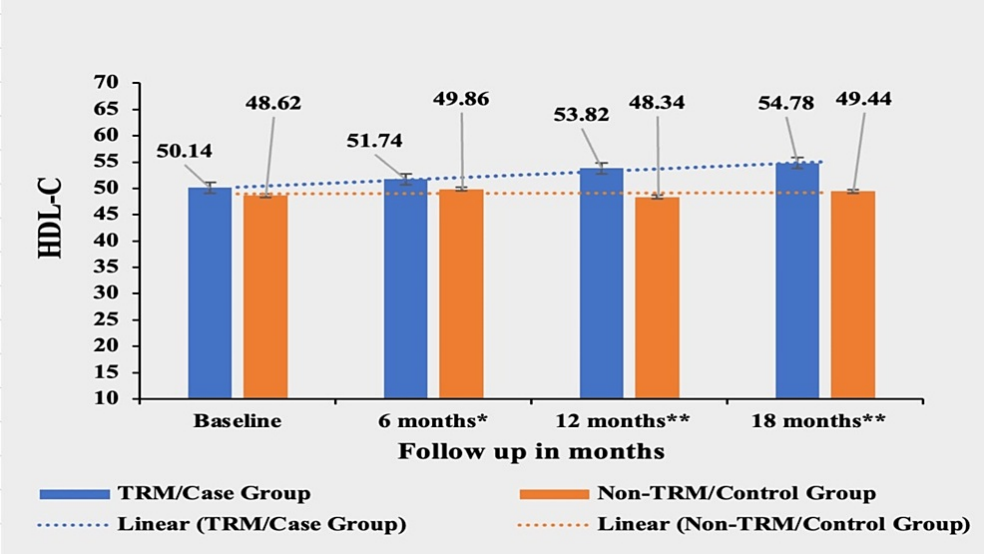
Comparative analysis between TRM/case group and non-TRM/control group on the basis of HDL-C Columns represent the mean values of HDL-C for TRM and non-TRM groups; error bars represent standard deviation (mean±SD) * p-value < 0.05; ** p-value < 0.0001 TRM: time-restricted meal intake, Non-TRM: non-time restricted meal intake HDL-C: high-density lipoprotein cholesterol

Patients in the TRM group experienced a significant increase of 9.25% in HDL-C from their baseline value of 50.14±8.58. The mean value of HDL was 51.74±27.5 in the first follow-up, 53.82±32.1 in the second follow-up, and 54.78±27.2 in the third follow-up. In contrast, the non-TRM group showed a smaller increase of 0.82% from their baseline value of 48.62±9.31 with a mean value of 48.62±9.31 in the first follow-up,48.34±18.6 in the second follow-up and 49.44±23.5 in the third follow-up. The mean of HDL-C was not significantly different at the baseline p value 0.3791 between the two groups, but the difference became highly significant in subsequent follow-ups, signifying a greater increase in HDL-C in the case/TRM group. The p-value was <0.0249 at the first follow-up, <0.0001 at the second follow-up, and < 0.0001 at the third follow-up (Figure 3).

Low-Density Lipoprotein (LDL)

**Figure 4:**
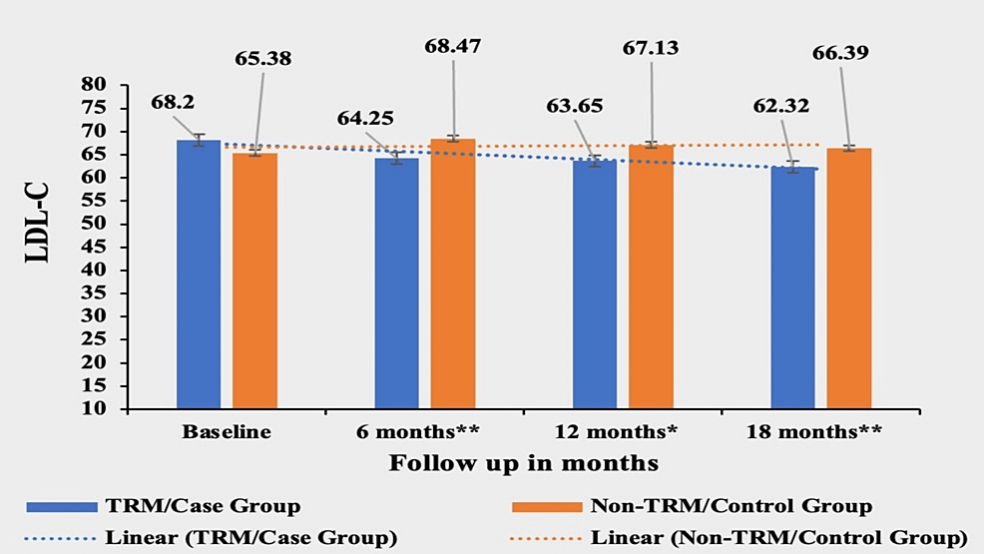
Comparative analysis between TRM/case group and non-TRM/control group on the basis of LDL-C Columns represent the mean values of LDL-C for TRM and non-TRM groups; error bars represent standard deviation (mean±SD) * p-value < 0.001; ** p-value < 0.0001 TRM: time-restricted meal intake, non-TRM: non-time restricted meal intake; LDL-C: low-density lipoprotein cholesterol

Patients in the TRM group experienced a significant decrease of 8.62% in LDL-C from their baseline value of 68.20±16.2. The mean value of LDL was 64.25±13.8 in the first follow-up, 63.65±12.7 in the second follow-up, and 62.32±12.2 in the third follow-up. In contrast, the non-TRM group showed a small increase of 1.54% from their baseline value of 65.38±19.3 with a mean value of 68.47±17.74 in the first follow-up,67.13±19.2 in the second follow-up and 66.39±20.3 in the third follow-up. The mean of LDL-C was not significantly different at the baseline between the two groups (p-value of 0.5943) but the difference became highly significant in subsequent follow-ups, signifying a greater reduction in LDL-C in the case/TRM group: p-value was <0.0002 at the first follow-up, <0.0034 at the second follow-up and <0.0002 at the third follow-up (Figure 4).

Very Low-Density lipoprotein (VLDL)

**Figure 5:**
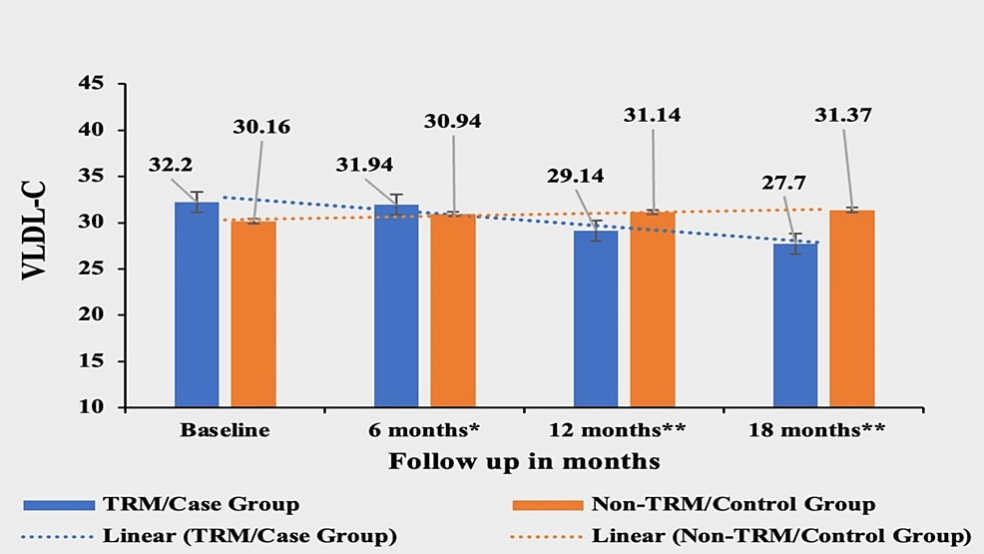
Comparative analysis between TRM/case group and non-TRM/control group on the basis of VLDL-C Columns represent the mean values of VLDL-C for TRM and non-TRM groups; error bars represent standard deviation (mean±SD). * p-value < 0.05; ** p-value < 0.0001 TRM: time-restricted meal intake, Non-TRM: non-time restricted meal intake; VLDL-C: very low-density lipoprotein cholesterol

Patients in the TRM group experienced a significant decrease of 13.97% in VLDL-C from their baseline value of 32.20±18.7. The mean value of VLDL was 31.94±27.6 in the first follow-up, 29.14±17.5 in the second follow-up, and 27.70±37.1 in the third follow-up in the TRM group. In contrast, the non-TRM group showed a small increase of 4% from their baseline value of 30.16±24.2, 30.94±13.9 in the second follow-up, 31.14±34.0 in the third follow-up and 31.37±22.1 in the third follow-up. The mean of VLDL-C was not significantly different at the baseline between the two groups, (p-value of 0.5526) but the difference became highly significant in subsequent follow-ups, signifying a greater reduction in VLDL-C in the case/TRM group: p-value was <0.0233 at the first follow-up, <0.0001 at the second follow-up and <0.0001 at the third follow-up (Figure 5).

## Discussion

Our study investigated the effects of TRM on fasting lipid profile in patients with T2DM over an 18-month period. Our results demonstrated a notable decrease of TC and LDL-C within the TRM group. The decrease in TC by 14.17% and in LDL-C by 8.62% from their baseline levels is particularly compelling when juxtaposed with the minimal reductions observed in the non-TRM group. The impact of TRM on plasma lipid levels appears to vary considerably across studies. While significant decreases in TG levels were observed in a few TRM studies [17-21], no significant changes in TG levels were noted in other studies [6,10,22-31]. Intriguingly, in two iso-caloric trials, fasting TG concentration actually increased [32,33]. Regarding cholesterol levels (TC, HDL, and LDL), the evidence regarding improvements through TRM is inconsistent. While most TRM interventions did not result in significant changes in cholesterol levels, some interventions showed improvements. These improvements included decreases in TC and LDL [31,21] or notable increases in HDL blood concentrations [28,21]. However, in one study, the TRM protocol led to an increase in TC and LDL cholesterol levels [34]. The differences in observations in various studies could be due to the difference in the duration of eating and fasting windows which differed from trial to trial and due to disparate cohorts being observed; the participants ranged from healthy, resistance-trained men in the study by Moro et al. [20] to adults with metabolic syndrome in the study conducted by Wilkinson et al [31].

Similar to its influence on glucose metabolism, TRM appears to affect lipid metabolism irrespective of weight loss or caloric restriction [21,33,34]. Research indicates that the effects of TRM on glycemic control appear to be less variable compared to its effects on plasma lipid profiles. One potential explanation for this observation could be attributed to the diurnal rhythm of glucose tolerance, which typically decreases from morning to evening and night. In contrast, lipid circadian rhythms tend to exhibit considerable inter-individual variability, which may contribute to the variability in the effects of TRM on plasma lipid profiles [35]. Balancing blood lipid levels stands as a paramount factor influencing blood glucose regulation among patients with T2DM [36]. Furthermore, blood glucose levels are linked to an elevated risk of obesity, which serves as a determinant of lipid or lipid ratio parameters [37]. Dyslipidemia in diabetes involves various quantitative and qualitative abnormalities in lipoprotein metabolism; these affect the acceleration of atherosclerosis. Elevated TG levels and low HDL are quantitative abnormalities. Qualitative abnormalities manifest as an elevation of minute, dense LDLs) and large VLDL1 [8]. Other abnormalities involve increased TG content in LDL and HDL, glycation of apolipoproteins, and increased vulnerability of LDL to oxidation. Kinetic abnormalities are characterized by elevated VLDL1 production, reduced VLDL catabolism, and increased HDL catabolism. Although LDL-C levels may appear normal, LDL particles have reduced catabolism, leading to atherogenesis in T2DM. Resistance to insulin hormone and its deficiency, which are hallmarks of T2DM, are seemingly responsible for the pathogenesis of diabetic dyslipidemia. Insulin plays a crucial role in regulating lipid metabolism, and disruptions in insulin signaling pathways can lead to dyslipidemia. Additionally, certain adipocytokines for example, retinol-binding protein 4 and adiponectin may also play a role in diabetic dyslipidemia. Overall, diabetic dyslipidemia represents a complex interplay of metabolic abnormalities that significantly increase the risk of cardiovascular complications in individuals with T2DM. Management strategies targeting dyslipidemia are essential for reducing cardiovascular risk and improving overall health outcomes in these patients [38]. TRM's beneficial effects on dyslipidemia stem from its harmonization with circadian rhythms and biological clocks, influencing glucose management, beta cell responsiveness, body composition, weight regulation, oxidative stress reduction, and metabolic flexibility.

A study by Khan et al. conducted in Islamabad among healthy individuals with 16 hours of TRM and a feeding window of eight hours for 26 days found that TG (p = 0.032), TC (p = 0.008), and LDL (p = 0.004) showed significant alteration, although there was no significant alteration in VLDL (p = 0.117). Specifically, total TG decreased from 104 to 100 mg/dl and from 89 to 95 mg/dl, TC decreased from 152 to 141 mg/dl and from 155 to 133 mg/dl, and LDL decreased from 104 to 93 mg/dl and from 102 to 87 mg/dl. Additionally, VLDL decreased from 21 to 19 mg/dl and from 19 to 18 mg/dl. Conversely, total HDL increased from 27 to 28 mg/dl and from 28 to 31 mg/dl for both female and male samples, respectively [39]. These results corroborate the findings observed in the TRM group of our study as in our study TC decreased from 168 to 144.2 mg/dl, TG decreased from 145.4 to 109.4 mg/dl, LDL decreased from 68.2 to 62.32 mg/dl in the TRM group with minimal change in the control group. All the above values changed significantly and in contrast to Khan et al.’s study, VLDL in our research also registered a significant decrease from 32.2 to 27.7 mg/dl. This finding of improved lipid profile in TRM protocol is important as Khan et al.’s study and our study both were conducted in Southeast Asian populations and could illustrate the beneficial effects of TRM, especially in this population cohort.

Interesting effects on lipid profile parameters were seen on increasing the fasting window through the early time-restricted feeding (eTRF) protocol which is a dietary strategy in which food intake occurs earlier in the day and thereby, the length of the overnight fast is extended. A seven-week study by Karras et al. [32] followed overweight individuals for seven weeks in which the eating window was from 08:00 am to 4:00 pm with 16-hour fasting; additionally, a five-week study by Sutton et al. studied male pre-diabetic individuals who had a six-hour eating window before 3 pm and 18-hour fasting. Both studies reported an increase in TG levels with eTRF [33]. In contrast, a two-week crossover study by Hutchison et al. in obese males with an eating window from 08:00 am to 5:00 pm and 15-hour fasting showed a decrease in TG levels following eTRF [19]. 

A study was conducted by Kesztyus et al. among healthy males for two weeks wherein the eating window was from 08:00 am to 4:00 pm. In this study, 16-hour fasting and eTRF did not significantly affect TG levels, despite observing a decrease in body weight after eTRF intervention [25]. In the study by Karras et al., the eTRF group displayed a decrease in HDL-C, which is typically considered anti-atherogenic [32]. Conversely, Sutton et al. found an increase in TC relative to the control group, although this relative increase in TG and TC was attributed to a decrease in these variables in the control group [33]. Notably, concentrations of TG and TC in the eTRF arm remained unchanged after the study relative to baseline levels. These variations underscore the complexity and inconsistency in the effects of eTRF on lipid profiles, highlighting the need for further research to elucidate these findings.

There have been studies that have examined the role of the number of meals per day on lipid profile parameters and it was found that consumption of nine meals per day led to a reduction in TC and LDL-C by 10% and 14%, respectively; however, eating six meals per day resulted in a decrease in total cholesterol by 1% to 8% and LDL cholesterol by 6% to 8% [40-42]. Conversely, eating three meals per day showed minimal to no change [38,43-45]. Intriguingly, in a controlled feeding study, 23 participants who consumed all of their daily energy needs in a single sitting experienced increases in TC and LDL-C of 19% and 25%, respectively, post-eight weeks [46]. Cumulatively, these findings suggest that increasing the frequency of meal intake might decrease cholesterol levels while reducing the number of meals taken might have detrimental results. In contrast with the findings above we observed in our study that patients in the TRM group experienced a significant decrease of 8.62% in LDL-C from their baseline value of 68.20±16.2. In contrast, the non-TRM group showed a small increase of 1.54% from their baseline value of 65.38±19.3. We can provisionally, state that decreasing the eating window could lead to significant improvements in the fasting lipid profile of T2DM patients. Meal frequency during the day in our study was limited to three meals, namely breakfast, lunch, and dinner with two snacks in between. We had not fixed the calorie intake of patients but the frequency of meals in all our patients averaged out at three meals per day. We were able to observe encouraging results in the various parameters of lipid profile even without increasing or decreasing the meal frequency in our patients, suggesting that the fasting period could potentially have a bigger role to play than the number of meals per day.

Studies have noted the effect of increasing or decreasing the number of meals typically on HDL and in a randomized, crossover trial, participants experienced a notable 17% increase in HDL-C levels from baseline when they consumed all of their energy requirements within a single daily meal over an eight-week period [46]. In contrast, HDL-C levels remained unchanged when participants consumed three meals per day. Notably, this change in HDL cholesterol was independent of the macronutrient composition of the diet, as dietary cholesterol and fatty acid intake remained constant. Altering meal frequency to more frequent intervals, ranging from 6-17 meals per day, did not significantly affect HDL cholesterol concentrations [38,41-45]. These results suggest that modifying meal frequency within iso-caloric conditions has minimal impact on HDL cholesterol levels. Despite these findings, the exact reason behind the observed increase in HDL cholesterol with a reduction in meal frequency to one meal per day remains unclear and warrants further investigation. Our study demonstrates an increase of 9.25% in HDL-C over a period of 18 months which is a statistically significant change and it improves the overall prognosis for a patient of T2DM. HDL-C is cardioprotective in nature and this particular parameter could change the profile of a patient from diabetic dyslipidemia to a normal lipid profile. Our study has made the observation that in T2DM patients, even without fixing the caloric intake and without rigidly fixing the frequency of meal timing, an increase in HDL could be obtained over long periods of time.

The beneficial effects of TRM on lipid parameters in our study could be due to TRM's synchronization with circadian rhythms and biological clocks, influencing glucose regulation, beta cell responsiveness, body composition, weight control, oxidative stress mitigation, and metabolic switchability. Also, recent studies have shed light on the association between gut bacterial diversity and lipid blood profiles, revealing links between fasting sugar levels, total TG, TC, LDL, and reduced gut bacterial diversity compared to control cohorts. These findings highlight the intricate interplay among gut microbiota composition, metabolic health, and dietary interventions such as intermittent fasting [47]. The circadian rhythm of many genes involved in lipid metabolism is disrupted in diet-induced obesity (DIO), but TRM can restore their cyclical expression [47]. One potential mechanism by which TRM affects host metabolism is by modifying the gut microbiome. TRM reinstated cyclical variation in the Lactobacillus family, which typically exhibits cyclical patterns in mice fed a normal chow diet but not in those with DIO. Certain Lactobacillus species have been linked to conditions such as diabetes and obesity. These species possess bile salt hydrolases, which can modify gut luminal bile acids and influence bile acid signaling. Moreover, TRM also restored the Ruminococcaceae family, which includes genera like Oscillibacter, hypothesized to offer protection against the metabolic consequences of obesity [47].

Overall, TRM appears to be a promising dietary strategy for improving metabolic health, particularly in overweight individuals. However, further research is warranted to better understand its mechanisms of action and optimize its implementation for different populations and metabolic conditions. Our study provides valuable insights into the potential benefits of TRM in managing T2DM, particularly among Indian patients, over an 18-month follow-up period. The significant decrease in lipid profile observed among TRM adherents underscores the efficacy of this feeding regimen and the potential of TRM as an adjunctive therapy alongside conventional medications for comprehensive diabetes management. Importantly, the absence of adverse effects in study participants emphasizes the safety profile of TRM, likely attributed to stringent adherence to selection criteria. However, the successful adoption of TRM necessitates careful guidance, motivation, and counseling by healthcare professionals, particularly during the initial phase of implementation.

Limitations

Several potential confounding factors may impact our study results. Firstly, the use and dosage of statins provided to patients, regardless of group allocation, represent a significant confounder. The medications in prescribed doses were provided to all patients irrespective of whether they were in the TRM group or the control group. No patient was denied the standard conventional treatment of T2DM. Secondly, genetic factors contribute to variations in lipid profiles among patients and may influence outcomes. Additionally, the intrinsic reduction of dyslipidemia with improved glycemic control in T2DM patients poses another confounding factor. Still, we have addressed this confounding factor because most of our patients had sufficient control of their blood sugar. Dietary intake of saturated fats, which vary among patients, presents another potential confounder. General instructions to reduce the use of saturated fats in diet were given to all patients irrespective of their group in order to minimize the effect of this factor. Lipids profile testing was done in fasting conditions as the level of TC and TG increased in the prandial state. Lastly, the level of physical activity among patients could also impact study outcomes. We did not ask patients enrolled in the study to start or leave any physical activity that they were already doing, thereby we tried to reduce the role of physical activity as a confounding factor. While efforts were made to address these factors, their potential influence cannot be entirely eliminated.

It's important to acknowledge that TRM may not be appropriate for certain demographic groups due to various considerations. These groups include children and adolescents who are still in the active growth stage, individuals reliant on medications or insulin injections to manage blood sugar levels (especially those with type 1 diabetes), individuals dealing with acute illnesses, eating disorders, pregnant or breastfeeding women, and those with severe kidney or liver diseases or cancer. For these populations, TRM might pose potential risks, and therefore, any contemplation of TRM should involve consultation with and supervision by a healthcare professional. Furthermore, there are notable research gaps and unanswered questions in the realm of TRM that necessitate further investigation. A significant limitation is our reliance on self-reported dietary intake by the patients. Due to resource constraints and the large number of participants involved, it was impractical to monitor all 273 patients closely for the entire 18-month duration of the study. Instead, we maintained regular communication with the patients through phone calls, emails, and messages at intervals of seven to eight days. However, adherence to the TRM regimen primarily depended on the patients' willingness and commitment, which introduces potential errors and variability in the data. Self-reporting of dietary intake is susceptible to recall bias and inaccuracies, as individuals may unintentionally misreport their food consumption. Moreover, the level of detail and accuracy in reporting may vary among patients. These limitations in dietary assessment could have influenced the interpretation of the results and the overall effectiveness of the TRM intervention.

To address these limitations in future studies, more rigorous monitoring methods such as direct observation or the use of wearable devices for dietary tracking could be employed. Additionally, strategies to enhance adherence and compliance to the prescribed regimen should be explored, such as providing regular counseling, support, and incentives to patients.

## Conclusions

In light of these findings, TRM emerges as a safe, convenient, and straightforward dietary strategy, not only for individuals managing T2DM but also for those seeking effective weight loss methods. Looking ahead, further research involving diverse patient populations, personalized TRM protocols based on individuals' chronotypes, and exploration of additional health parameters will be crucial for validating and expanding upon the positive outcomes observed in our study. These endeavors hold promise for advancing diabetes care and promoting better health outcomes in the future.
